# Co-developing an intervention to facilitate safe and early transition to neonatal home care for very preterm infants: a mixed-method study evaluating the impact of patient and public involvement

**DOI:** 10.1186/s40900-025-00775-3

**Published:** 2025-08-15

**Authors:** Sofia Arwehed, Ylva Thernström Blomqvist, Elin Carlsson Filipowicz, Anna Axelin

**Affiliations:** 1 Department of Women’s and Children’s Health, Uppsala University, Uppsala, Sweden; 2 Center for Research and Development in Gavleborgs County Council, Gävle, Sweden; 3 Department of Nursing Science, University of Turku, Turku, Finland; 4 Patient and Public involvement Coordinator, Stockholm, Sweden

**Keywords:** Co-designed intervention, Discharge, Neonatal home care, Infant premature, Patient and public involvement.

## Abstract

**Background:**

Very preterm born infants face elevated risks of adverse neurodevelopmental outcomes, with prolonged hospitalisation associated with poorer cognitive, motor, and language development. Contributing factors include limited parental presence, insufficient stimulation, and exposure to stressful procedures. In Nordic countries, neonatal home care programmes support early discharge by enabling parents to manage nasogastric tube feeding at home under specialist supervision. However, inconsistent discharge practices delay the transition to home by creating parental uncertainty and making the process more vulnerable to staff discontinuity. This study aimed to co-develop an intervention to support safe and early discharge and evaluate the impact of engaging parents and healthcare professionals as collaborators throughout the research process.

**Methods:**

A descriptive mixed-methods study with an embedded process evaluation was conducted guided by participatory action research methodology. A Steering Committee consisting of two parents, a neonatal nurse, a researcher, and a coordinator managed the process. Five parents and seven healthcare professionals from three Swedish neonatal units representing diverse care models were purposively recruited for creative workshops, ensuring diversity in gender, culture, and professional background. Patient and public involvement (PPI) was evaluated through anonymised impact log surveys, a process log, standardised meeting minutes, semi-structured interviews with Steering Committee members, and a written survey of public contributors. Field notes, post-it notes, mind maps, and audio recordings supported data validation.

**Results:**

The co-development process resulted in an intervention tool designed to visualise the neonatal care journey, discharge criteria, infant development, and parental preparation, including milestones to track progress and strengthen parental roles. More than 90% of stakeholder recommendations were implemented, closely aligning the tool with family needs. Key enablers of meaningful collaboration were a respectful, emotionally safe environment and a shared commitment to collaborative decision-making. Paired reflection supported individual expression. Parents appreciated the opportunity for emotional processing, while professionals valued gaining deeper insight into family perspectives.

**Conclusion:**

This study demonstrates the feasibility and value of PPI in developing a neonatal care intervention. The resulting tool is intended to enhance predictability, standardisation, and timely discharge preparation while strengthening the parental role. A forthcoming feasibility study will assess its potential to improve discharge practices, support parental well-being, and facilitate safe and early transition to home.

**Trial registration number:**

279,523 (Registered 28th of September 2023 in Researchweb, Region of Gävleborg domain).

**Supplementary Information:**

The online version contains supplementary material available at 10.1186/s40900-025-00775-3.

## Abstract (plain language summary)

Parents of very preterm born infants often feel stressed when it’s time to take their infant home from the neonatal unit. Many have described the discharge process as unpredictable and lacking clear structure. To help address these challenges, we carried out a research project in partnership with parents and healthcare professionals. Together, we developed a new approach to better support families in preparing for discharge and making a smoother, earlier transition to neonatal home care.

The project also aimed to help researchers better understand the impact and value of working closely with parents and healthcare professionals. To do this, we collected feedback through surveys, interviews, meeting notes, and logs. Our analysis showed that over 90% of the suggestions from parents and professionals were included in the final tool, making it well aligned with families’ needs. A respectful group atmosphere and shared purpose were key to meaningful collaboration. Parents appreciated the emotional support and sense of voice the process gave them, while staff valued the insight into family experiences.

This study shows that working together with parents and professionals is not only possible but also improves the relevance and usefulness of the final tool. A future study will explore whether the tool helps families feel more prepared and confident when going home.

**Supplementary Information:**

The online version contains supplementary material available at 10.1186/s40900-025-00775-3.

## Key Message

• Prolonged hospitalisation is associated with poorer neurodevelopmental outcomes in very preterm infants, while inconsistent discharge practices contribute to increased parental stress and delayed transition to home care.

• A mixed methods study was conducted to co-develop an intervention supporting safe and early discharge, involving parents and healthcare professionals as active collaborators throughout the research process.

• The resulting tool visualises the neonatal care journey, discharge criteria, infant development, parental preparation, and milestones aiming to strengthening the parental role.

• Over 90% of stakeholder recommendations were incorporated, and enablers of collaboration included emotional safety, shared decision-making, and paired reflection.

• This study demonstrates the feasibility and impact of PPI in developing a neonatal care intervention. A forthcoming feasibility study will evaluate the tool’s potential to improve discharge practices, support parental well-being, and facilitate timely transition to home.

**Supplementary Information:**

The online version contains supplementary material available at 10.1186/s40900-025-00775-3.

## Background

Long hospitalisation have been identified as an independent risk factor for poor neurodevelopmental outcomes in very preterm infants [1, 2]. A study by Kellner et al. [1] found that poorer cognitive, motor, and language outcomes at 1–2 years corrected age were associated with higher postmenstrual age at discharge, after adjusting for medical risk factors. They concluded that inadequate parental presence and holding, lack of age-appropriate stimuli, and exposure to painful or stressful procedures contribute to a negative hospital environment. Acknowledging this, Lundberg et al. [3] and Moen et al. [4] suggests discharge should occur as early as medically safe.

In the Nordic countries, neonatal home care involves parents managing nasogastric tube feeding and establishing oral feeding for clinically stable infants at home, as described by Rosenbaek et al. [5] Despite their clinical stability at discharge, these infants remain high-risk and require specialised neonatal care. [5] Family-centred care practices and generous parental leave policies in the Nordic countries create favourable conditions for a gradual transition of care responsibilities to parents [6, 7]. This approach facilitates safe discharge [3, 5] and reduces the length of stay [8]. However, our previous study on discharge practices in the Nordic region [9] identified a need for improved standardisation and earlier initiation of discharge preparation. A subsequent Swedish study [10] found limited parental involvement in care planning with the transition to home being staff-driven and delayed due to staff discontinuity [10]. 

Becoming a parent includes emotional bonding, interpreting the infant’s signals, meeting their needs, and advocating for their well-being [11]. Having a very preterm infant brings significant complexity to this process [12, 13]. Along with the trauma of often unexpected preterm birth, lack of knowledge about care routines and infant development can cause anxiety and feelings of helplessness [14, 15]. Sudden deterioration of the infant’s condition is common, and healthcare professionals (HCPs) competence is crucial for the infant’s well-being. These factors impact parents’ adaptation to their new role and heighten their sensitivity to change [16, 17]. Thus, preparation for transition from the hospital to home must be timely, consistent, and inclusive of parents’ entire experiences [15, 18].

Adding parents’ perspective and exploring their needs is essential to understand how to improve care [19, 20]. There are several examples of patient and public involvement (PPI) in neonatal research [21, 22], and reviews describing both facilitators and barriers for successful collaboration [23–25]. Growing recognition of the value of PPI in developing complex interventions implies that stakeholder’s perspective should be included in each phase [26]. By involving parents and HCPs throughout the research cycle [27, 28], with shared decision-making and priority setting [24, 29, 30], interventions can be designed to meet real needs [20, 31] and reduce research waste [24, 32]. 

Evaluation of PPI is equally important to gain understanding of its impact on intervention research, clinical outcomes and implementation [33–36]. Robust evaluation tools are lacking [37], but using tools and principles from existing frameworks and structured reporting of PPI [38], can increase knowledge on how to conduct effective collaborative research [39]. 

This study aimed to develop an intervention to facilitate safe and early transition to neonatal home care and evaluate the impact of involving parents and HCPs as collaborators in this process.

## Methods

### Objectives and design

This was a descriptive mixed-methods study with a concurrent, embedded process evaluation. The objectives were to: (1) Describe the contributions of parents and HCPs throughout the research cycle; (2) Evaluate the influence of stakeholders’ voices and impact on the intervention; (3) Assess the relevance of the intervention to stakeholders’ needs; and (4) Explore parents’ and HCPs’ experiences of collaboration.

A PPI plan was developed, guided by the study-focused [39] frameworks of Shippee et al. [40] and the National Institute for Health Research (NIHR) [41]. The PPI process was documented using adapted versions of the NIHR Impact log [42] and the PIRIT tracking tool [43], with additional guidance from Boivin et al. [34] Reporting followed the Critical Outcomes of Research Engagement (CORE) by Dillon et al., [35] and Guidance for Reporting Involvement of Patients and the Public (GRIPP2) long form checklist by Brett et al. [44] Participatory design methods, used in workshops, were inspired by Clemensen et al. [21, 45].

### The intervention development process

In May 2024, the SC held a start-up meeting to establish rapport, discuss the research question and aim, and identify the needs of families transitioning to neonatal home care. The projects scientific background and the rationale for PPI were presented. The SC collaboratively developed a structure for activities, decision-making, and documentation. Following the meeting, the SC conducted a study visit to the University Hospital in Odense, Denmark, to exchange experiences on participatory design methods with another research group [20, 46]. During the visit, materials from the start-up meeting were categorised, analysed, and refined in relation to the study aim to identify potential intervention components. Brainstorming sessions used post-it notes and mind maps, and the process was documented through individual field notes.

Over the next eight months (excluding a one-month summer break), the SC met digitally (online) 1–3 times per month for one-hour sessions. The intervention development process was guided by the Medical Research Council’s framework for developing complex interventions [26]. The SC reviewed relevant literature and compared findings with the lived experiences of parents and HCPs. This process informed the programme theory, logic model, and identification of key intervention components. Communication between meetings was maintained via WhatsApp and email. To broaden perspectives and validate priorities, the SC decided to conduct creative workshops with additional PCs.

In January 2025, a two-day creative workshop was held with the PCs. Participants received pre-workshop information and were encouraged to take field notes. The workshop began with an icebreaker led by a parent, followed by an introduction to the study. Participants were divided into two groups: one for parents, facilitated by SC parents, and one for HCPs, facilitated by SC HCPs. Four guiding questions structured the sessions: (1) What facilitates safe and early discharge? (2) What is important for the well-being of preterm infants and their parents in this transition? (3) How can these needs be addressed through an intervention? (4) How can the intervention be refined to facilitate implementation?

**Fig. 1:**
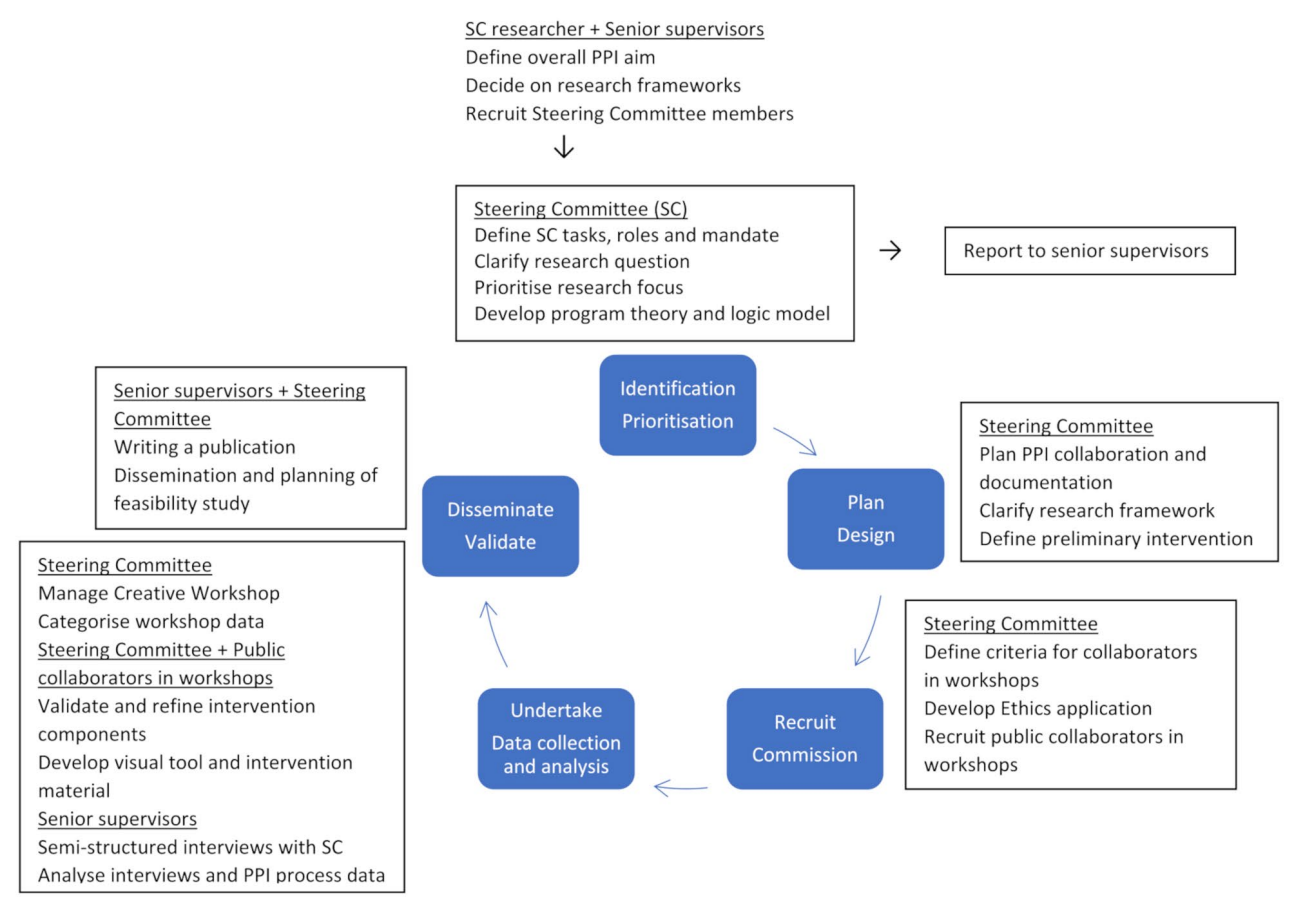
Research cycle and PPI collaboration in the development of an intervention to facilitate very preterm infants transition from hospital to neonatal home care. **Abbreviations:** PC, Public Collaborators in workshop; PPI, Patient and Public Involvement SC, Steering Committee

Each session began with individual brainstorming using post-it notes, followed by in-depth group discussions. The groups later reconvened to share findings, ask follow-up questions, and further develop ideas. All written materials were collected and photographed. After the workshop, the SC reviewed and categorised all materials into core intervention components. A preliminary visual tool was designed by an SC parent and presented at a digital (online) half-day workshop, where remaining questions were addressed and uncertainties resolved. Figure 1 illustrates the intervention development process in relation to the research cycle.

### Setting and participants

Stakeholders, in this study parents and healthcare professionals, were engaged as collaborators throughout the research cycle and all phases of intervention development (Fig. 1). A Steering Committee (SC) was established to manage the research process, comprising the first author (researcher and physician) and four PPI representatives. Two parents and one assistant nurse were recruited by the neonatal home care team at Uppsala University Hospital, while a third parent, appointed as PPI coordinator, was referred to the research team after expressing interest at a social event. Three senior researchers (including the second and last authors, both registered nurses) supervised the study and received regular progress updates, while all decisions were made within the SC. The PPI plan, including roles, priorities, and incentives, was guided by the SUPPORT Patient and Public Engagement Planning Template [47] and NIHR guidelines [48]. The SC was responsible for defining research questions, refining the study design [24, 32], assist with recruitment [40], and guiding intervention development, including the creation of a logic model [26].

In addition, five parents and seven HCPs from one Level II and two Level III neonatal units were recruited as public collaborators (PCs) for a two-day creative workshop and a half-day digital (online) workshop (Fig. 1). Participants were selected from a pool of 17 candidates nominated by participating units, with attention to gender balance, multiprofessional representation, and cultural diversity. The final composition was affected by a late cancellation. Stakeholder characteristics are presented in Table 1.

**Table 1:** Characteristics of members of the steering committee and public collaborators

Characteristics	Parents (*N* = 8)	HCPs (*N* = 9)
**Role in project**		
Steering Committee	3 (60)	2 (40)
Public Collaborators	5 (42)	7 (58)
**Gender**		
Female	4 (50)	8 (89)
**Age (years)**		
25–34	7 (87)	3 (33)
35–44	1 (13)	0 (0)
45+	0 (0)	6 (67)
**Ethnic background**		
Non-Nordic origin	1 (13)	2 (22)
**Profession**		
Registered nurse	–	3 (33)
Physician	–	3 (33)
Assistant nurse	–	3 (33)
**Years in neonatal care**	–	
> 10	–	6 (67)
**Parental status**		
First-time parent	6 (75)	–
Higher education	5 (63)	–
**Infant’s GA at birth**		–
< 25 weeks	3 (37)	–
25–27 weeks	4 (50)	–
> 28 weeks	1 (13)	–
**Infant characteristics**		
BPD	3	–
IVH III-IV	1	–
ROP III	1	–
GI surgery	2	–
**Time since discharge, months**		–
< 6	4 (50)	–
6–12	3 (37)	–
> 12	1 (13)	–
**Model of home care experienced**		
Home visits	4 (50)	–
Outpatient visits	4 (50)	–
phone support 24/7	8 (100)	–

n (%). Abbreviations: BPD, bronchopulmonary disease; GA, gestational age; GI, gastrointestinal; HCP, Healthcare professionals; IVH, intraventricular haemorrhage; ROP, retinopathy of the premature

All parents and healthcare professionals in this study received written and oral information and gave their informed consent to participate.

### Data collection for evaluation of PPI

Data collection for evaluating PPI was integrated throughout the intervention development process, using multiple complementary tools: (1) Impact log survey: An anonymised survey with Likert-scale and open-ended questions, adapted from the People in Health West of England PPI Impact Log [42]. A link to the survey was emailed to SC members after each meeting. (2) Process log: Adapted from the PIRIT tracking tool [43], this log documented recommendations, actions, decisions, and their initiators (parents, HCPs, or researcher). It was maintained by the researcher (SA) during meetings. (3) Meeting minutes: Standardised minutes recorded individual contributions, documented by the PPI coordinator, and reviewed for accuracy at the end of each meeting. (4) Semi-structured interviews: Conducted with SC members by an external researcher using a guide (Supplement S1) developed by the first and second authors (SA, YTB), based on the NIHR framework [41]. (5) Written survey: Distributed to public collaborators (PCs), using open-ended questions from the interview guide (Supplement S2).

Anonymous field notes were collected from all SC members during the study visit, SC meetings, and workshops to provide context for the collaborative experiences. Meetings and workshops were audio-recorded to validate the Process log and meeting minutes.

### Data analysis

To enable a systematic integration of multiple data sources and gain a comprehensive understanding of stakeholder perspectives, we employed the “following a thread” method developed by Moran-Ellis et al. [49] This approach is suitable for mixed-method studies, as it allows the researcher to trace key analytical insights across datasets and weave them into a coherent narrative. Initially, each dataset was analysed separately (methods detailed below) to identify key categories, themes, and analytical questions requiring further exploration. Impact Log data were analysed using descriptive statistics for Likert-scale responses and thematic analysis for open-ended responses (Table 3). Process log data were coded and categorised using a deductive thematic approach based on the CORE framework [35] (Table 2). Recommendations and decisions were quantified and cross-checked against meeting minutes to validate stakeholder contributions. Semi-structured audio-recorded interviews were transcribed verbatim and analysed by the second and last authors (AA, YTB) using qualitative content analysis as described by Graneheim and Lundman [50]. Meaning units were identified, coded, and sorted based on their similarities and differences in relation to the study aim, then abstracted into subcategories and categories. Written survey responses from PCs were analysed in the same manner by the first author (SA).

In the following integration, categories and themes identified in one dataset were traced across the others to form coherent threads [49], creating a constellation of findings and generating a multi-faceted understanding of stakeholders’ experiences of collaboration and the intervention development process. During this iterative and cyclic process, the researchers raised and discussed analytic questions, generating new threads to follow. Findings were discussed among the authors until consensus on the analysis and final interpretation was achieved. When uncertainties arose, the original data were revisited for cross-validation.

## Results

### The intervention – a visual tool to facilitate a safe and early transition to home

The PPI process resulted in the development of a visual tool designed to enhance predictability, ensure timely parental preparation, and support parents in adapting to their new role. The final intervention includes this tool and a 1–2-hour instructional session for HCPs, aiming to facilitate a safe and early transition to home-based neonatal care for very preterm infants and their parents.

The intervention supports the transition by visualising (1) the care journey and standard procedures, (2) expected infant development and discharge criteria, (3) information and parental preparation, (4) key milestones, and (5) individualised support. Desired outcomes for parental well-being include an increased sense of coherence and empowerment, and reduced levels of stress and depressive symptoms. Key factors promoting infant well-being include increased parental presence and involvement in care, both of which are associated with improved physiological and developmental outcomes, as well as reduced length of hospitalisation.

The intervention tool consists of three graphic sheets: Infant TO HOME, Parent TO HOME, and AT HOME.

Infant TO HOME includes the categories (1) Eat & Grow, (2) Skin & Temperature, (3) Breathing, (4) Standard examinations, and (5) Medications. Parent TO HOME includes the categories (1) Milestones, (2) Well-being, (3) Information, (4) Education, and (5) Consultation. Each category features QR codes with hospital-specific information.

AT HOME covers aspects of neonatal home care and follow-up, including skin-to-skin care, hygiene, breastfeeding, weaning from tube feeding, and access to digital (online), physical, and psychological support at home.

### Contributions of the steering committee and public collaborators to the research process and intervention development

Over 90% of PPI recommendations were implemented during the research process, influencing research priorities, planning, management, and intervention design (Table 2). Many ideas were expressed and shared within the SC. The members repeatedly returned to the aim, both to prioritise and choose a path forward, and to ensure they did not lose sight of what was central to the intervention. All participated in decision-making; however, it was not clear to the members whose idea was whose, but they felt that they had contributed to them during the research process. The amount of potential influence was greater than they expected.

*“We had more influence than I thought we would have.” Mother*.

PCs primarily contributed during the intervention design phase. By sharing their experiences and perspectives, the SC described how PCs helped validate stakeholder needs and priorities previously identified by the SC. During the intervention development process, recommendations from parents and HCPs provided a continuous ‘red thread’ from identification of needs and programme theory to the final design of the intervention. Critical outcomes of PPI are detailed in Table 2.

**Table 2:** Impact of steering committee and public collaborators’ engagement on the research process and final intervention (based on process log data, thematically categorised according to the critical outcomes of research engagement CORE; Dillon et al. [35])

Recommendations from Steering Committee and Public Collaborators’ influencing the research process and their effects on intervention development				
Recommendations made by	**Identification**	**Prioritisation**	**Designing**	**Planning/Management/** **Undertaking**
Steering Committee	5/5 (100)	35/35 (100)	42/46 (91)	64/65 (98)
Public collaborators in workshop	─	6/6 (100)	139/155 (90)	─
Critical outcomes of PPI by research stage	*Validated research question*: Investigate how to facilitate - Safe and early transition to home. - Infant and parent well-being during the transition. *Identified needs*: - Increased predictability - improved timing in parental preparation. - Support in the parental role.	*Prioritised components for a safe and early transition home*: A visualised care journey with a standardised approach; - Discharge criteria for infants and parents. - Standard procedures. Early information and continuous preparation Acknowledging the preceding trauma in becoming a parent and its impact on the transition into the parental role.	*Design recommendations for the intervention*: Develop a combined roadmap and checklist tool designed to visualise - The care journey and standard procedures. - Infant development and discharge criteria. - Information and parental preparation. - Milestones to support parental role and instill hope. - Individual support when needed. Designed primarily for parents. Accessible from admission and supported by HCPs bedside and during consultations. Disseminated by parents and HCPs.	*Recommended actions to facilitate research planning*,* management*,* and undertaking*: - Social activities; contributing to an open group climate. - Define roles and expectations. - Repeat the red-thread; the *what* and the *why* to keep the scope of the project. - Provide time to clarify and discuss before ‘research decisions. - Have someone to reflect with between meetings. - Validate SC input with additional PCs; for intervention to be relevant to many. - Keep it simple and repeat; words, structure, information material - Use SC moderators in workshops to reduce hierarchy and facilitate contribution.

Numbers are recommendations acted on/total number of recommendations (%). Abbreviations: HCP, Healthcare Professionals; PPI, Patient and public Involvement; PC, Public Collaborators in workshop; SC, Steering Committee

### Steering committee and public collaborators’ voice and impact on the intervention

#### Parent perspective at the centre

All members of the SC clearly expressed that the driving force in the research project was to value parents’ perspectives in the intervention development process. This was realised in action, as they considered that the parents’ voices were strong throughout the project.

With the parents’ voices at the centre, HCPs acquired a more profound understanding of parents’ experiences, which guided decision-making in both SC meetings and workshops. Therefore, according to the participants, the project advanced many things that were important to parents, not those that the HCPs considered important for parents.

*“When parents can think and speak freely*,* different things come into focus. When they are given the mandate to say what they think and not just answer our questions.” Physician*.

#### All perspectives had an impact

At the same time, the HCPs contributed valuable medical insights. In SC meetings, the parent perspective was balanced and refined with the HCPs perspective. According to the members of the SC, both perspectives were needed to understand and achieve the goal.

This process was also evident during workshops, which HCPs described as *‘a learning experience’* and parents as *‘being heard and sharing with others who understand’*. Both SC members and PC noted the surprisingly different perspectives parents and HCPs could have about the same situation. These “aha” moments occurred both in smaller SC meetings and during workshops when the groups reconvened, allowing for dialogue and follow-up questions. For them, this underscored the importance of bringing all perspectives together.

Parents and HCPs also described how the workshops facilitated reflection on intercultural perspectives, such as perceptions of sibling visits in the unit and communication barriers due to lack of interpretation. These insights influenced the intervention design, including the use of images, QR codes, and the need for translated materials.

#### Paired reflection helped to express voice

Having opportunities to discuss and reflect on ideas with another SC member between meetings helped develop and express ideas during meetings. One SC member expressed that missing this opportunity decreased her contribution to discussions within the SC. During workshops, spontaneous pairings emerged shaped by shared experiences of parenthood, gender, profession, or cultural background. Both parents and HCPs reflected on how the group’s composition contributed to power balance, facilitating the expression of diverse perspectives.

### The relevance of the intervention to stakeholders’ needs

Both parents and HCPs emphasised that the intervention should be primarily designed to meet parents’ needs. Early in the PPI process, key priorities were identified: increasing predictability, improving the timing of parental preparation, and strengthening the parental role. These priorities were collaboratively translated by the Steering Committee and public contributors into specific components that informed the design of the intervention tool (Table 2). All parent-prioritised needs and corresponding recommendations were incorporated into the final intervention.

#### A visualised care journey

Parents highlighted the need for a visible care journey from the point of admission, outlining expectations for both infant development and parental involvement. They recommended a checklist with discharge criteria for infants and parents to encourage and empower in active participation in their infant’s care.

*“When you arrive at the unit*,* you want to know what is required for you to go home.” Father*

These insights guided the design of the intervention tool, which combines a visual roadmap and checklist to make the infant care journey more transparent and predictable. Being able to reflect on the next steps and initiate discussions, rather than solely relying on healthcare professionals’ initiative, was described as supportive and empowering in the transition. HCPs also recognised the importance of strengthening parents’ voices and supported the view that the care journey should reflect milestones prioritised by parents, both in infant development and parental involvement.

Feeling safe in leaving the neonatal unit was seen as essential, for parents bringing their infant home and for HCPs ensuring a secure discharge.

*“I think we should visualise clear criteria*,* those needed to be checked before care can continue safely at home.” Mother*

#### A standardised approach

A universally embraced suggestion was that the intervention tool should be based on the infant’s developmental maturity rather than gestational age. While recognising the need to tailor care to each infant and family, both parents and HCPs found it helpful for structuring information and managing changes throughout the hospital stay.

*“The standard approach will be to sit down with the family at admission and create an individualised plan for what is most likely for this particular infant. And when needed revise the plan*,* together” Physician*.

Collaboration between the neonatal home care team and the neonatal unit were functioning well and recommended to remain in place. Continuity of care, with the same HCPs supporting the family in both hospital- and neonatal home care settings, was seen as particularly valuable.

Both HCPs and parents emphasised that the intervention tool was intended to complement, not replace, consultations. While bedside healthcare professionals were identified as the primary users, physicians were also recognised as playing a critical role in implementation. Parents considered their involvement essential for defining and reinforcing goals and ensuring a consistent and supportive transition.

#### Early information and continuous preparation

Parents described feeling overwhelmed by the volume of written information and reported a lack of understanding regarding standard procedures. In response, healthcare professionals suggested adding QR codes to the intervention tool to provide families with early information in an accessible format. Both groups emphasised the importance of providing all families with repeated information, regardless of language barriers, during the early phase of hospitalisation.

Parents appreciated being actively involved in their infant’s care, such as in tube feeding and oxygen regulation. They expressed a desire for HCPs to encourage early hands-on involvement while recognising and respecting parents’ initial lack of confidence and fear of harming their infant.

*“I remember how hard it was when they told me in the first few weeks that you know your baby best and I thought: I don’t!” Mother*

These components were incorporated to help parents gradually build knowledge, safely meet their infant’s needs, and gain confidence in taking responsibility for their infant’s care.

*“Taking responsibility is part of being a parent!” Father*

#### Trauma preceding transition

Due to the trauma associated with preterm birth, parents described an initial fear of making decisions that might harm their infant. This could lead them to prefer that HCPs made decisions on their behalf, even regarding their own involvement in their infant’s care.

*“In the beginning I wanted HCPs to be more in control*,* instead of giving parents a choice. For example*,* when they asked me if I wanted to sleep with my child*,* I didn’t know what to say. Is it good or bad? What should I do?” Mother*

Parents also described how even small changes in their infant’s condition could undermine their confidence in themselves and in the HCPs. Visualising progress across developmental areas was therefore essential, particularly when advancement in a specific area was lacking. One mother reported how achieving certain milestones helped ease her anxiety by indicating reduced risk of complications. Based on this input, parents and HCPs mutually designed the intervention to reflect progress in both infant development and parental competence.

#### Transition into parent role

Parents emphasised the importance of HCPs introducing and supporting activities that parents normally do with their infant. All described key situations when they began to see their infant not only as a patient but as their infant, often triggered by ‘normal’ activities such as putting on baby clothes or going for a walk. These moments were viewed as turning points in the parent–infant relationship and in the transition of becoming a ‘normal’ parent. After a discussion, these activities were incorporated to promote bonding and, by normalising, instil hope for the family’s future.

*“The first walk was very important for us. Going out together.” Father*

Another key aspect was to celebrate progress through awarding diplomas, writing a diary, or taking photos to make milestones meaningful and health progress visible. To support implementation, parents jointly composed a supplemental text describing these recommendations, where they explained how these hope-inspiring activities, combined with planning for an early transition home, helped them feel motivated and mentally prepared.

*“Each milestone you pass creates well-being. The more milestones you complete*,* the better you feel.” Father*

#### Consensus on design reflecting key priorities

After the two-day workshop and a follow-up SC meeting, a SC parent designed a prototype for the intervention tool. This was presented at a final digital (online) workshop with PCs, where content and design received strong support. The tool and accompanying instructional material were reviewed against the needs and priorities identified throughout the SC and workshop process. Both parents and HCPs expressed confidence that they would work well in clinical practice.

*“I would have really appreciated having this during our time in the neonatal unit.” Parent*

### Steering committees and public collaborators’ experience of collaboration

#### The importance of group climate

The SC held eighteen meetings, and the summarised Impact log response rate was 65/81 (80%). The entire SC agreed or strongly agreed that it was easy for themselves and for others to contribute during meetings (Table 3).

**Table 3:** Steering committee’s summarised response to the impact log likert scale questions. Reported as n/total numbers (%)

Impact log Likert scale questions	I strongly disagree	I disagree	Neutral	I agree	I strongly agree	Not available
I found the meeting format suitable for its purpose.	0/0	0/0	0/0	0/0	65/65 (100)	─
I found it easy to contribute.	0/0	0/0	0/0	6/65 (9)	57/65 (88)	2/65 (3)
I found it easy for everyone to contribute.	0/0	0/0	0/0	4/65 (6)	59/65 (91)	2/65 (3)

Analysis of the summarised responses to the open-ended Impact log questions revealed that the primary factor was the open, respectful group climate, which fostered a sense of security. Another important facilitator was the mutual commitment to the research topic, making contributions and dialogue meaningful. Barriers to contribution primarily stemmed from time constraints and interruptions due to clinical work or family presence during meetings

#### Collaborative and respectful engagement

Across all data sets, both parents and HCPs described a sense of collaborative partnership and mutual respect, which supported idea generation and enabled in-depth discussions. Both groups emphasised that hearing diverse voices and reflecting on each other’s perspectives brought new insights into the needs and choices of both parents and HCPs. Active participation was encouraged throughout the project, and collaborators worked together to ensure that everyone had the opportunity to contribute.

*“I have never felt*,* so to speak*,* unheard. Instead*,* it has always been very*,* well*,* very appreciative and very open*,* with a great atmosphere.” Mother*

#### Shared vision

The shared vision of a safe and early transition home fostered meaningful collaboration and supported decision-making. Parents reported feeling encouraged to express their needs and contribute suggestions, while HCPs focused on identifying strategies for effective implementation. Both groups described a shared commitment to collaborative decision-making and active participation, which they perceived as empowering. They valued their role in driving meaningful change and expressed a collective commitment to supporting the dissemination of the intervention.

*“The main reason we said yes to participating is because it’s awful to be in the neonatal unit! And if you can do even the smallest thing to improve the circumstances*,* both for the children and the families*,* then it’s absolutely worth it.” Mother*

#### Personal development

Parents emphasised the emotional aspect of participating in the project and valued the opportunity to reflect on their care journey, something several had not previously done. They described sharing their perspectives and listening to other parents and HCPs as part of an emotional recovery process.

*“This journey with our son has deeply affected me. And I would say that this project has helped me gain valuable insights.” Mother*

HCPs reflected on the personal and professional impact of PPI, finding it both rewarding and transformative. Parents’ stories provided new clinical insights, even for experienced professionals. Several instances where parental needs and priorities had been misunderstood were reported, which HCPs described as ‘wake-up calls.’ This fostered a sense of community among collaborators, with both parents and HCPs expressing a desire to continue working on PPI initiatives.

#### Facilitation and support structures

The early-phase study visit to Denmark facilitated cohesion among SC members, as they spent time together and shared both personal lives and project ideas. Everyone described how this early bonding enhanced productivity, and comfort in sharing opinions and experiences in subsequent meetings. The SC researcher was a driving force in the project and had initially a key role in building open group dynamics and facilitating exchange of ideas and thoughts. There was no sense of power imbalance in the group, making it easier to express opinions and disagree when necessary. It was also beneficial to have a little push to voice one’s thoughts aloud.

*“I don’t feel this imbalance of power*,* so to speak*,* and I think it’s also important to say what you think and dare to disagree.” Mother*

During workshops, SC parents noted that PCs were new to PPI and roles were initially more traditional. The method of having separate group discussions followed by mutual reporting and reflection helped overcome power barriers. It also revealed when needs and experiences differed between parents and HCPs. Parents’ suggestions, such as including ‘clothing’ as a milestone and encouraging parents to leave the unit to recuperate together, raised objections from HCPs. Another recommendation to appoint a family contact person was deemed unfeasible by HCPs due to resource constraints. The PPI coordinator and a SC parent both highlighted that complex questions took time but appreciated the shared willingness to listen to everyone’s views.

In the small SC, roles were well distributed, and the work was effective. The researcher maintained methodological rigor while fostering an open atmosphere. Members appreciated delegating final research responsibility to the researcher, though the SC collectively kept the project on track. SC members found it beneficial to have both in-person and online meetings, depending on the task and project phase. Since discussions were valued and all decisions were made within the group, the meeting agenda and schedule had to be flexible. Nonetheless, to meet deadlines and ensure progress, the group collectively imposed limits on discussions. During workshops, time limitations proved more challenging. Both parents and HCPs reflected on the need to respect the therapeutic process among parents.

## Discussion

Incorporating parents’ perspectives is essential for optimising neonatal care [19, 20]. In a recent study by Thivierge et al., [51] 248 parents of extremely preterm born infants were asked: *“Knowing what you know now*,* what do you wish doctors would have told you about prematurity before and/or after your child’s birth?”* The main recommendations focused on improving communication regarding (1) preparation for discharge and life after the NICU in a stepwise, personalised, and practical manner (40%), and (2) the provision of practical, functional information about parenting in the NICU throughout the clinical trajectory (35%). Our study addresses these gaps and further demonstrates the impact of PPI, with over 90% of parent-informed recommendations being implemented, significantly shaping research priorities and intervention design. By centring parental voices and allowing time for dialogue and reflection, the intervention was aligned with families’ needs. Moreover, healthcare professionals and researchers gained valuable insights into parents’ lived experiences.

Very preterm infants and their families should be transitioned to home-based neonatal care as soon as it is safely possible, given the adverse effects of prolonged hospitalisation [1–4].  A systematic review and meta-analysis by Hamer et al. [52] demonstrated that early supported transfer interventions can reduce hospital stay without increasing readmission rates. In our study, parents identified key shortcomings in current discharge practices, including a lack of predictability and unclear criteria for determining infant and parental readiness for discharge. In response, parents and healthcare professionals co-developed an intervention aimed at addressing these gaps and empowering parents to take an active role in discharge preparation, thereby facilitating earlier transitions home. Healthcare professionals noted that implementing this intervention may enhance standardisation and reduce delays associated with staff discontinuity, as observed in Sweden and other Nordic countries [9, 10].

While these outcomes are promising, Skivington et al. [26] emphasise the importance of not only identifying stakeholder priorities but also understanding the underlying reasons for those priorities. Transition is a complex process, influenced by both internal and external factors [53]. Becoming the parent of a very preterm infant affects nearly every aspect of daily life and heightens sensitivity to change [17]. Klawetter et al. [54] advocate for embedding tailored mental health support throughout the care trajectory, with particular attention to the discharge transition. This was echoed by parents during SC meetings and workshops, where they shared experiences of suspended parenthood, expressed the need for emotional support both during hospitalisation and post-discharge, and described the PPI process itself as therapeutic. By visualising accessible support and milestones relevant to family well-being, and celebrating progress throughout the care journey, the intervention may strengthen parents’ sense of coherence and help reduce stress and depressive symptoms. The effectiveness of the co-developed intervention remains to be evaluated in clinical practice.

This study applied established PPI frameworks and data collection tools to co-develop a clinical intervention and to evaluate and report the impact and experiences of PPI. Agyei-Manu et al. [19] emphasise, such approaches are important for advancing knowledge on effective collaboration methods. Both parents and HCPs identified an open, respectful group climate and a shared vision for clinical improvement as key enablers of successful collaboration. Reported benefits of the PPI process were consistent with those described by Agyei-Manu et al. [19] and Shen et al.: [55] parents reported a sense of empowerment and achievement in contributing to meaningful change; healthcare professionals gained new insights; and researchers observed improvements in the quality and relevance of the intervention. A challenge related to PPI was the extended duration of research-related decisions and group discussions, which exceeded initial expectations. This was not formally measured but was observed by the research team and attributed to the need to accommodate parents’ therapeutic processes and emotional engagement.

Prioritising parents’ perspectives was a key aspect of the intervention development process. Although both parents and HCPs contributed equally to and endorsed the intervention design, the specific needs of healthcare professionals may have been underrepresented. This could potentially influence the intervention’s acceptability and implementation in clinical practice. By definition, participatory action research seeks to foster collaboration between researchers and stakeholders to drive meaningful change [56]. A forthcoming co-designed feasibility study will assess the applicability of the intervention in clinical practice, as well as its potential short- and long-term effects on infant and parental outcomes.

## Conclusion

This study demonstrated the feasibility and impact of PPI in the development of a neonatal care intervention. The resulting intervention tool was designed to enhance predictability, standardisation, timely preparation, and support for the parental role. The findings contribute to the growing evidence base on effective PPI practices and highlighted the value of co-production across all phases of the research cycle in advancing family-centred neonatal care. A forthcoming feasibility study will explore whether the intervention may improve discharge practices for very preterm infants, support parental well-being, and contribute to a safer and more timely transition to home.

## Supplementary Information

Below is the link to the electronic supplementary material.

Supplementary Material 1

Supplementary Material 2

## Data Availability

The datasets used and/or analysed during the current study are available from the corresponding author on reasonable request.

## Abbreviations

HCP: Health Care Professionals
NIHR: National Institute for Health Research
PC: Public Contributor
PPI: Patient and Public Involvement
SC: Steering Committee
