# Supplementary material for: Co-developing an intervention to facilitate safe and early transition to neonatal home care for very preterm infants: a mixed-method study evaluating the impact of patient and public involvement
Source: Res Involv Engagem. 2025 Aug 15;11:97. doi: 10.1186/s40900-025-00775-3 (PMC12357328; doi:10.1186/s40900-025-00775-3)
Supplement: Supplementary file 1 — Supplementary Material 1 [file 40900_2025_775_MOESM1_ESM.pdf]

UPPSALA
UNIVERSITET

Interview guide TO HOME Study III

Co-developing an intervention to facilitate safe and early transition to home care for very preterm infants: A mixed-method study evaluating the impact of Patient and Public Involvement.

Information to participants

We aim to evaluate the method and the impact of collaboration between researchers, parents, and staff in the development of the intervention. This interview seeks to gain a deeper understanding of what has facilitated or been a barrier to collaboration within the project, how you have experienced your own and others' contributions, and what you perceive as the likely effects of conducting the project in partnership with those directly affected by healthcare.

Introduction

Could you briefly describe your overall experience of participating in the project?

Key discussion points and guiding questions:

- *Could you describe how you understood the project's purpose and goals?*
- *What have you found to be the most important aspect of the project?*
- *Has anything surprised you during the course of the project?*
- *How do you feel the involvement and contributions of parents and healthcare staff have influenced the nearly completed intervention?*
- *How would you describe your role in the project and your ability to influence its direction?*
- *What has helped you to contribute to the project?*
- *What has made it more difficult for you to contribute?*
- *Is there anything else you would like to add?*
