# Supplementary material for: Co-developing an intervention to facilitate safe and early transition to neonatal home care for very preterm infants: a mixed-method study evaluating the impact of patient and public involvement
Source: Res Involv Engagem. 2025 Aug 15;11:97. doi: 10.1186/s40900-025-00775-3 (PMC12357328; doi:10.1186/s40900-025-00775-3)
Supplement: Supplementary file 2 — Supplementary Material 2 [file 40900_2025_775_MOESM2_ESM.pdf]

UPPSALA
UNIVERSITET

Co-developing an intervention to facilitate safe and early transition to home care for very preterm infants: A mixed-method study evaluating the impact of Patient and Public Involvement.

Survey aim

This survey aims to explore how collaboration between researchers, parents, and healthcare professionals influenced the development of the intervention. We seek to understand what supported or challenged the collaboration, how you experienced your own and others' contributions, and what impact you believe this approach may have had on the project.

Questions

(Please write your thoughts for each question, starting with the question number, e.g., 1. I feel/think that...)

1. *Briefly describe your overall experience of participating in the project.*
2. *What are your thoughts about the purpose and goals of the project?*
3. *How did you perceive your role in the project?*
4. *How would you describe the collaboration within the project?*
5. *In what ways do you think the involvement of parents and healthcare professionals influenced the intervention?*
6. *What do you feel was the most important aspect of the project?*
7. *Is there anything else you would like to share with us—or with others interested in involving parents and healthcare professionals in research to improve care?*
